# Full recovery after non-target cerebral embolization of n-butyl-cyanoacrylate occurred during emergency treatment of a facial arteriovenous malformation

**DOI:** 10.1186/s42155-019-0063-3

**Published:** 2019-06-29

**Authors:** Ezio Lanza, Nicolò Gennaro, Dario Poretti, Laura Straffi, Simona Marcheselli, Marco Tramarin, Vittorio Pedicini

**Affiliations:** 1 Department of Radiology, Humanitas Research Hospital, Via R.L. Montalcini 4, 20090 Pieve Emanuele, MI Italy; 2 Training School in Radiology, Humanitas University, Via Rita Levi Montalcini 4, 20090 Pieve Emanuele, MI Italy; 3 Department of Neurology, Humanitas Research Hospital, Via A. Manzoni 56, 20089 Rozzano, MI Italy

**Keywords:** Stroke, Arteriovenous malformation, Bleeding, Glue, Cyanoacrylate, Catheterization, Embolization

## Abstract

**Background:**

Non-target embolization is a well-known complication of endovascular procedures for arteriovenous malformation. However, few reports have described non target encephalic embolization, detailing its temporal evolution.

**Case presentation:**

A 41-year-old man presented with a massive hemorrhage in the oral cavity due to an arteriovenous malformation involving the left hemiface and tongue. Under conscious sedation, selective angiography was followed by endovascular embolization with a mixture of n-butyl-cyanoacrylate-methacryloxy-sulfolane (NBCA-MS) with Lipiodol. The hemorrhage was successfully arrested, but the procedure was complicated with a reflux of embolic material from the right external carotid artery into the common carotid, caused by strong unexpected coughing. Non-target embolization was confirmed by emergency CT and subsequent MRI. After initial neurological impairment, the patient recovered fully and was discharged after one week. No sequelae were confirmed by 9-months follow-up with CT and MRI. We describe technical aspects, multimodality imaging, clinical presentation, and follow-up of this peculiar case.

**Conclusion:**

Endovascular embolization of AVM fed by the external carotid is at risk for non-target brain embolization and general anesthesia should be considered to prevent inadvertent movements and master the delivery of the embolic agent A small amount of Lipiodol / NBCA-MS may be fully tolerated by the brain matter and partially reabsorbed without permanent deficit.

## Introduction

Arteriovenous malformations (AVMs) are low-resistance vascular congenital abnormalities made of dysplastic arteries and veins arranged in a primitive reticular network. Due to their persistent proliferative ability, proper management is challenging and may even require a multidisciplinary approach (Lee et al, 2013; Donnelly et al., 2000; Lee et al., 2004; Lee & Bergan, 2002; Nakazawa et al., 2007). Endovascular embolization is used to reduce blood flow to the AVM prior to surgery and has been proven as safe, feasible and effective (Rosen & Contractor, 2004; Liu et al., 2014; Yakes, 2004; Raffi et al., 2007; Krajina et al., 1991; Dabus et al., 2017). However, over the last two decades several complications have been described especially in the emergency setting, including cast spillover, migration and non-target embolization (Lanza et al., 2014; Pelz et al., 1995; Chagla & Balasubramaniam, 2008; Naithani et al., 2013; Li et al., 2005; Krishnamoorthy et al., 2007). However, few reports have described encephalic non target embolization and its temporal evolution (Jayaraman et al., 2008; Sugiu et al., 2004).

**Fig. 1:**
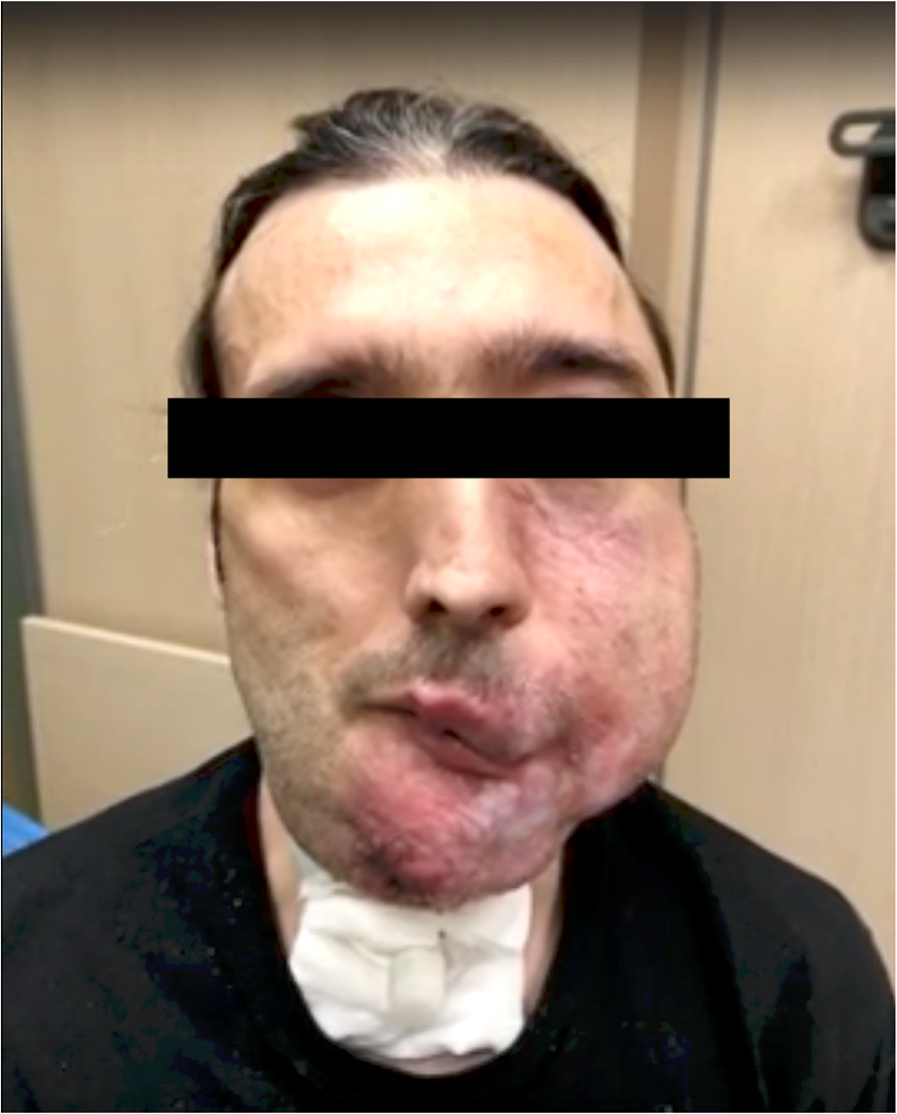
External presentation. Large arteriovenous malformation involving patient’s left hemiface and tongue

**Fig. 2:**
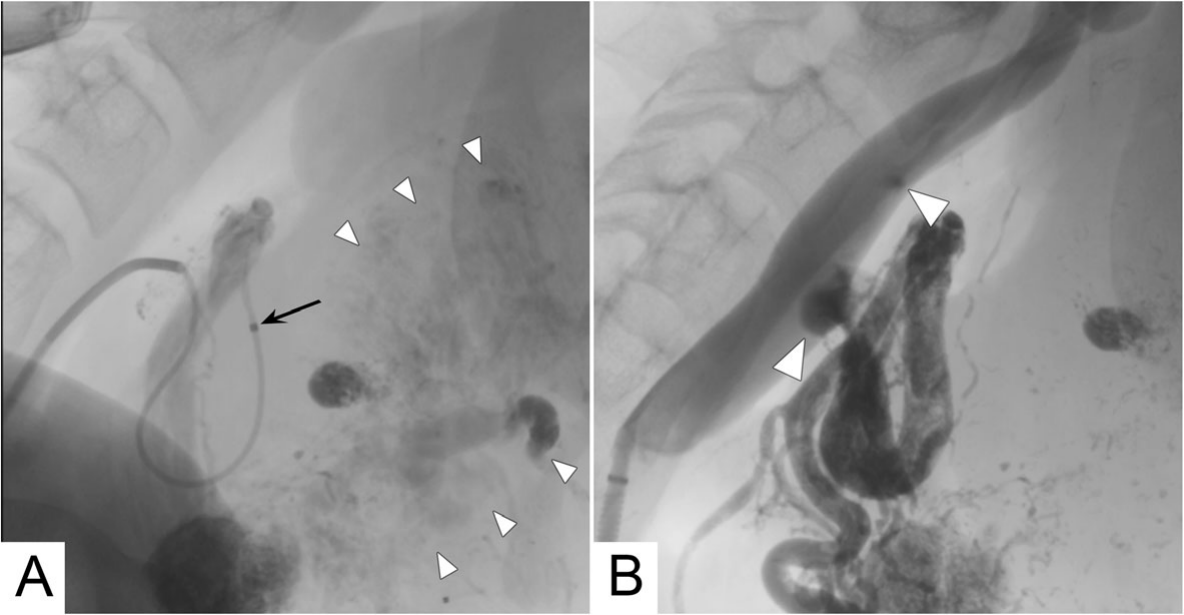
(**a**) Super-selective angiography of the AVM. Black arrow indicates the microcathetertip before embolization. Small white arrowheads outline the large facial AVM. (**b**) Control angiography from the common carotid after embolization. The microcatheter has been removed. White arrowheads indicate NBCA deposits in the common carotid, secondary to accidental reflux of the embolic agent during vigorous coughing

**Fig. 3:**
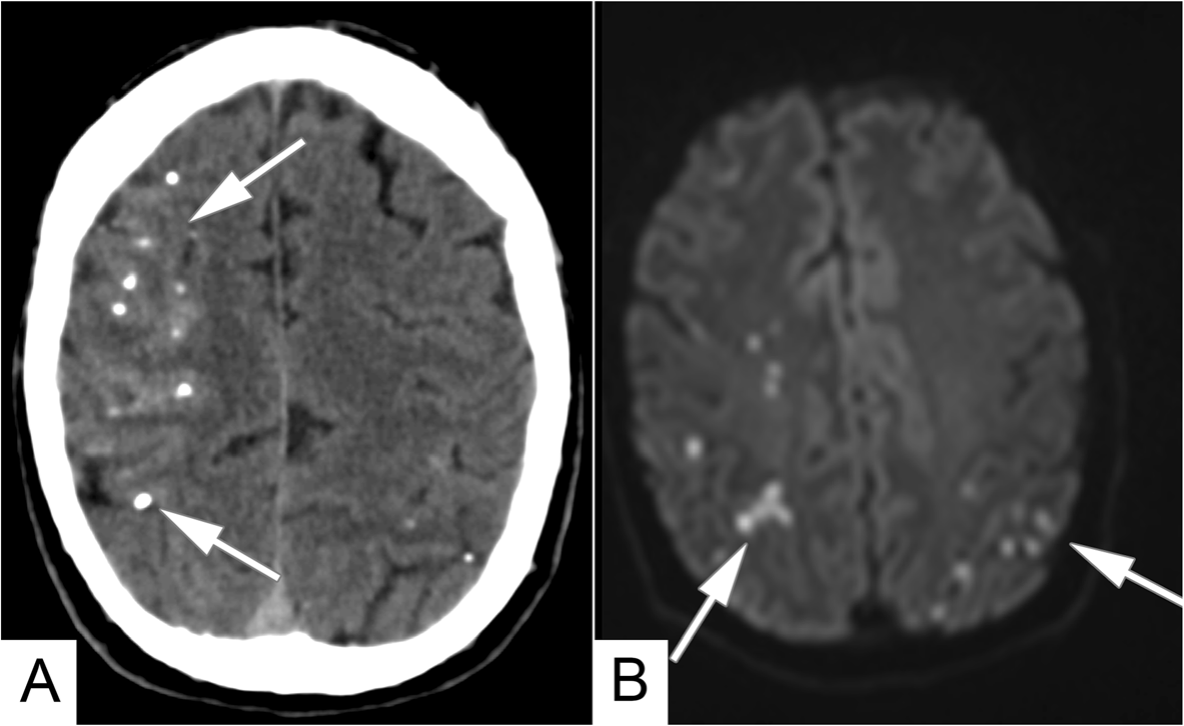
(**a**) Post-procedural non-enhanced axial CT. Multiple glue deposits in the right frontal and parietal lobes (white arrows). (**b**) Same day axial DWI MRI (b = 1000). of the brain showing acute infarcts both in right and left hemispheres

**Fig. 4:**
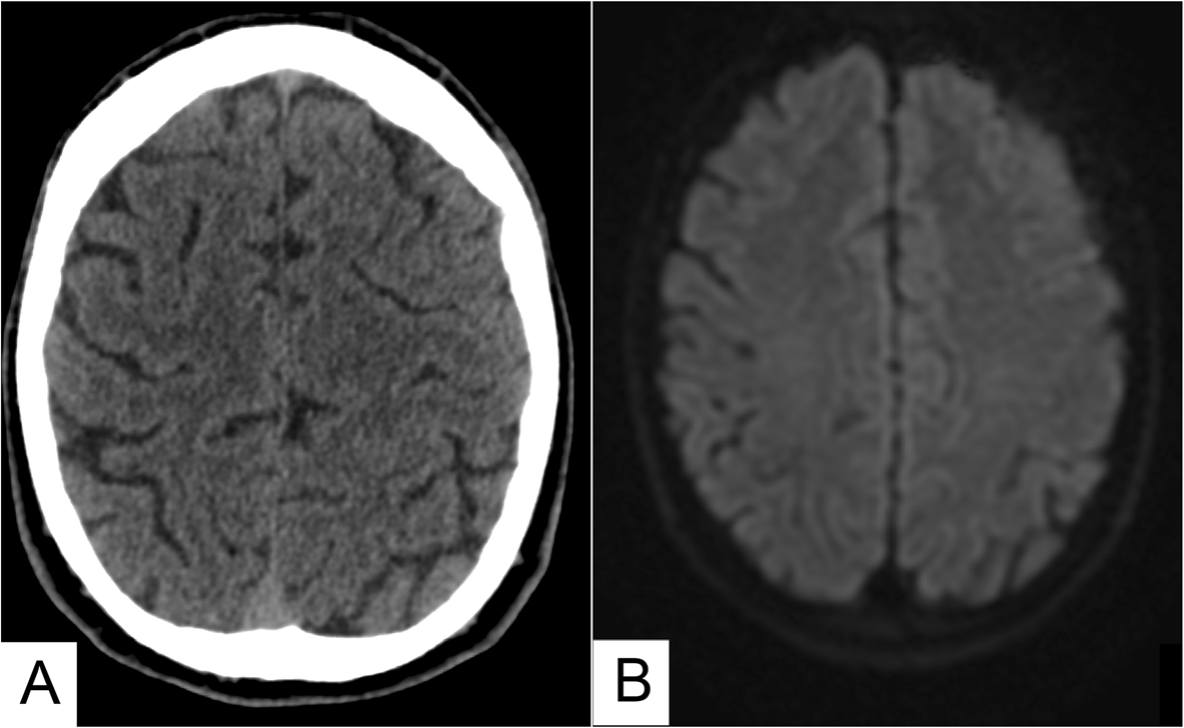
Nine months CT (**a**) and MRI (**b**) control. Complete disappearance of migrated material

In this report, we describe the event of non-target embolization of n-butyl-cyanoacrylate-methacryloxy-sulfolane (NBCA-MS) in the brain occurred during endovascular treatment of a facial AVM.

## Case report

A 41-year-old male with a history of recurrent bleeding from a left-sided facial AVM presented with massive hemorrhage in the oral cavity and decreased hemoglobin level from 11 to 9,9 g/dl in 24 h (Fig. 1). Under conscious sedation, angiography of the supra-aortic trunks showed nidal arteries originating from the left vertebral artery and the right external carotid. Patient’s airway was protected by the presence of tracheostomy.

The left vertebral artery was catheterized with a 0.027″ Renegade HI-FLO microcatheter (Boston Scientific, Marlborough, MA, USA) and a first super-selective embolization of the branches directed to the AVM was performed, using a 1:5 mixture of Glubran*®*2/Lipiodol (GEM, Viareggio, Italy/Guerbet, France). The compound was created by connecting and vigorously flushing two 10 mL syringes via a two-way stopcock, one syringe filled with 1 mL NBCA-MS and the other with 5 mL Lipiodol, until a homogeneous suspension was achieved. The right external carotid was then catheterized. Diagnostic angiography showed hypertrophic pathological nidal arteries directed to the AVM; the expected vascular anatomy was no more recognizable. The microcatheter was positioned far from the carotid bifurcation (approximately 7 cm) and deep into the nidus to lower the chances of retrograde embolization (Fig. 2a). The operator then started injecting the embolizing compound. Short before completing the filling of the nidus, the patient coughed vigorously causing an abrupt pressure increase and a temporary blood flow stoppage which led to partial reflux of the embolic agent into the common carotid (Fig. 2b). The reflux was also likely favoured by the increased pressure in the nearly embolized nidus.

Although the oral bleeding was successfully stopped, the patient started complaining of weakness in the left arm. He was promptly transferred to the emergency room to perform a non-enhanced CT of the brain, which showed multiple cortical and subcortical punctiform hyperdense areas caused by the migrated embolic material all over the frontal, parietal and occipital lobes in the right hemisphere, and also in the left parietal and occipital lobes (Fig. 3a). Same-day non-enhanced MRI with DWI sequences confirmed the presence of small acute infarcts (Fig. 3b). No alterations were seen in the cerebellar parenchyma.

The patient was transferred to the Neurology Unit where the clinical presentation was evaluated through the standard tools for assessment of stroke, the NIH Stroke Scale (NIHSS) and the Modified Rankin Scale (mRS). Ideomotor apraxia, weakness of left upper limb (NIHSS = 3) and inability in left hand daily dexterity (mRS = 3) were noted. The next day, patient clinical conditions improved: he was able to move the left limb (NIHSS = 1) and to use objects with left hand (mRS = 2). On the third postoperative day, there was significant improvement both of the upper extremity weakness (motor drift of the left arm resulted in NIHSS = 1) and of the distal weakness (mRS = 2).

One week later, no focal alterations could be detected using both stroke clinical assessment tools. The patient was finally discharged and continued his neurological rehabilitation at home.

One month after the procedure, neurological examination showed no recurrence and confirmed complete symptoms regression. Nine months later, the patient remained asymptomatic and both CT and MRI showed the complete disappearance of the embolic agent and the relative ischemic areas (Fig. 4a-b).

## Discussion

AVM are challenging conditions that may require emergency intervention when bleeding occurs in high-risk areas in contiguity to the main airway, like face and tongue. To achieve efficient embolization an ideal agent should travel distally from the point of injection and fill the whole nidus of dysplastic vessels, as opposed to occluding the supplying arteries alone (Yakes, 2004).

NBCA-MS is an embolic agent with large evidence of safety and efficacy in this and many other settings (Rosen & Contractor, 2004; Raffi et al., 2007; Pedicini et al., 2017). NBCA-MS is mixed with Lipiodol to make it radiopaque and to adjust its polymerization time. However, mainly due to its rapid polymerization and the inability to blend with other fluids, its behavior may prove unpredictable during injection and cause permanent damage if non-target embolization occurs (Lanza et al., 2014; Li et al., 2005). These risks should be foreseen by the operator and appropriate measures to avoid them should always be put in place. For instance, NBCA has been proven to induce coughing once deployed (Pelz et al., 1995), as it occurred in the present case; the use of deep general anesthesia could have prevented the reported complication, although the routine use of such an approach is not supported by any guideline or position paper. Other potential risk factors for failing the correct delivery of the embolic agent include the early retraction of the microcatheter during injection, lack of operator experience and highly diluted embolic compound.

NCBA-MS has shown several benefits compared to traditional cyanoacrylates and may be preferred for its slower polymerization time, which makes it more flexible. Moreover, the addition of MS has been reported to lower cytotoxic and inflammatory effect due to the milder exoteric traction (Loffroy et al., 2009; Loffroy, 2016).

Recent papers suggest that the deployment of Onyx Liquid Embolic System (Medtronic Neurovascular, Irvine, CA, USA) in an intracranial embolization setting correlates with a lower risk of neurological complications (Choo & Shankar, 2016). It is possible that Onyx’s high manageability due to its viscosity could have theoretically prevented the non-target embolization (Romero et al., 2009). Nevertheless, in an unstable setting of a not paralyzed patient under conscious anesthesia, the same feature could have also led to an early reflux into the common carotid artery during coughing, possibly with even more dramatic consequences considering the non-resorbability of all its components.

After reviewing the literature, where Onyx has been associated with higher costs, longer fluoroscopy times (Velat et al., 2008) and poorer visualization in case of reflux in small arteries (Katsaridis et al., 2008), our group is still endorsing the use of NBCA-MS. To overcome some limitations of Onyx, PHIL (MicroVention, Tustin, California) is a new generation non-adhesive liquid embolic agent comprised of a biocompatible polymer dissolved in dimethyl sulfoxide (DMSO) linked to an iodine agent, triiodophenol, for radiopacity purpose (Vollherbst et al., 2018).

In this adverse event our expectations about the evolution of the neurological status were far from optimistic since we expected permanent brain damage; instead, there was a complete resolution of the initial neurological impairment and radiological findings. This clinical condition, characterized by a NIHSS≤3, MRS ≤ 2 and initial positive DWI findings has been referred as minor stroke (Fischer et al., 2010). We suppose that, as opposed to the major stroke mechanism in which a large embolus blocks a proximal artery, we observed a more scattered and incomplete peripheral arterial embolization, which has probably allowed for compensation from adjacent vessels to take place (Romero et al., 2009). Consistently with the progressive neurological improvement, we may speculate that the majority of the displaced emboli was made of Lipiodol, which was more represented in the compound and is, unlike NBCA-MS, a slowly-resorbable embolic agent (Ethiodized oil - DrugBank, 2019).

## Conclusion

Embolization of AVMs with nidal arteries arising from the external carotid artery is at risk for non-target brain embolization. Patients’ immobility is desirable and deep anesthesia should be discussed with the anesthesiologist to prevent the coughing reflex, which can temporarily reverse blood flow and cause reflux. However, in this single case a small embolus of 1:5 mixture of NBCA-MS/Lipiodol was fully tolerated by the brain matter, leaving no permanent damage as confirmed after a follow-up of nine months.

## Data Availability

Further data and images regarding this clinical case are available upon request.

## Abbreviations

AVM: Arteriovenous malformation
CT: Computed Tomography
DWI: Diffusion Weighted Imaging
MRS: Modified Rankin Scale
NBCA: n-butyl-cyanoacrylate
NHSS: National Institute of Health Stroke Scale
